# Underrepresentation of ethnic minorities in hypertension research—a survey of enablers and barriers among South Asian and African communities in Glasgow

**DOI:** 10.1186/s13063-022-06542-z

**Published:** 2022-07-29

**Authors:** Stefanie Lip, Georgia Dempster, Sahil Jain, Katriona Brooksbank, Nazim Ghouri, Linsay McCallum, Sandosh Padmanabhan

**Affiliations:** 1 Institute of Cardiovascular and Medical Sciences, University of Glasgow, Glasgow, UK; 2 Department of Rheumatology and General Internal Medicine, Queen Elizabeth University Hospital, Glasgow, UK

**Keywords:** Blood pressure, Blood pressure measurement, Ethnic minorities, Hypertension, Public engagement, South Asian, African

## Abstract

**Background:**

Hypertension is the biggest contributor to the global cardiovascular burden with evidence for ethnic differences in treatment response and outcomes. Under-representation of ethnic minorities in clinical research is well known, and despite wide-ranging public engagement events by the Glasgow Blood Pressure Clinic team, there was a lack of participation of ethnic minorities in both engagement activities and clinical trials conducted by them. This study aims to explore the awareness and knowledge of hypertension and the facilitators and barriers to participation in hypertension clinical research among South Asian (SA) and African (AFR) communities in Glasgow.

**Methods:**

A survey questionnaire was co-developed with representatives from South Asian (SA) and African (AFR) patients and community members in Glasgow to understand awareness and knowledge of hypertension and enablers and barriers to participation in clinical research. The survey was distributed to adults (aged > 18) years of SA or AFR ancestry at public engagement events at venues that were frequently visited by these two communities in Glasgow.

**Results:**

The survey response rate was 337 (67.4%) consisting of 242 (71.8%) South Asian (SA) and 56 (16.9%) African (AFR) respondents. Thirty-nine questionnaires were excluded because of incompletion. Most of the respondents were not born in the UK and were in the 35–53-year group (AFR 29 (51%), SA 113 (47%)). The proportion living in the most deprived (SIMD 1) and least deprived (SIMD 5) was respectively 26 (12.4%) and 34 (16.2%) for SA and 20 (42.6%) and 2 (4.3%) for AFR. There was a considerable recognition that treatment needs to be ethnicity-specific (SA/AFR = 107 (48%)/23 (45.1%)) and that current cardiovascular disease treatment guidelines were not tailored for different ethnicities 84 (38.5%)/23 (45.1%). The key enablers encouraging research participation are enhanced health information, conducting aspects of their clinical research visits/appointments at a location they frequently visited and allowing a family member to accompany them. Barriers included concerns about the use of personal information and side effects of the new treatment.

**Conclusion:**

Our survey confirmed enablers and barriers to ethnic minority participation in research. We find improving and evolving awareness and beliefs among the ethnic minority population including community leaders. Thus, continual review of researchers’ beliefs and attitudes is also essential to ensure engagement activities keep up with these changing perceptions.

**Supplementary Information:**

The online version contains supplementary material available at 10.1186/s13063-022-06542-z.

## Background and rationale

It is well recognised among all stakeholders (researchers, clinicians, funders, policymakers) that ethnic minorities, despite constituting 1 in 8 of the UK population, are under-represented in clinical research [1, 2]. The consequences are wide-ranging. The limited generalisability of research findings [3] may have an adverse impact on resource allocation for services and research for these groups, thereby perpetuating and amplifying health inequalities with the continued marginalisation of these groups. In Scotland, ethnic minorities account for 2.7% (Asian) and < 1% (African) of the population, with a higher proportion in the cities Glasgow (15%), Edinburgh (8%), Aberdeen (8%) and Dundee (6%) [4]. Of the 15% of the population of Glasgow that belong to an ethnic minority group, 8.1% and 2.4% were of South Asian and African ancestries, respectively. Ethnic minorities in Scotland have lower mortality than the general population but a higher prevalence of cardiovascular disease and diabetes with poorer health outcomes [5]. In Scotland, over the last 15 years, considerable efforts have been made to understand and increase research involving ethnic minorities with a focus predominantly on diabetes and mental health. The Community Health Index (CHI) is a population register, which is used in Scotland for health care purposes. The CHI number uniquely identifies a person on the index. In an attempt to improve ethnicity information systematically and consistently in Scotland, the Retrocoding Project was initiated in 2009 that led to the inclusion of ethnic classification as part of the 2011 census and when linked to CHI numbers could potentially facilitate recruitment into trials through health service data [6]. However, in the Prevention of Diabetes and Obesity in South Asians (PODOSA) trial [7], investigators found that the recruitment of South Asian (SA) participants through the health service was neither efficient nor sufficiently effective, but this improved substantially with partnerships with local SA organisations and individuals and referrals by word of mouth from existing participants.

Hypertension shares several predisposing risk factors with diabetes and is considered a major cardiovascular and renal risk amplifier in diabetes, and strict BP control is one of the priority management targets in the diabetes treatment guidelines [8, 9]. Given the scale of health awareness and research engagement activities for diabetes in Glasgow, the expectation was a trickle-down effect on hypertension awareness and research. The Glasgow Blood Pressure Clinic (GBPC) team has been engaged in regular hypertension public engagement activities since 2012 through World Hypertension Day and May Measurement Month events with screening and educational activities conducted in supermarkets, bingo halls, community centres and public areas [10–12]. However, in 2019, the GBPC team was informed that its MRC-funded AIM-HY trial [12], which specifically on SA and AFR ethnic groups, was recruiting poorly. A review of other hypertension trials confirmed that there was almost no ethnic minority representation in all studies triggering this study to explore the awareness and knowledge of hypertension and the facilitators and barriers to participation in hypertension clinical research among South Asian (SA) and African (AFR) communities, the two largest ethnic minority groups in Glasgow.

## Methods

### Design of survey

A survey questionnaire was co-developed over several rounds of consultations and revisions with the NHS Greater Glasgow and Clyde patient engagement group, clinicians, trial research nurses and lay representatives from the SA and AFR groups. The survey was divided into three main sections: demographics, health and well-being, and clinical research (Additional file 1). The demographic section covered ethnicity, demographic data, education and socioeconomic status. The health and well-being section sought information on knowledge, awareness and individual assessment of the current medical conditions and health. This section included the assessment of awareness and understanding of hypertension and diabetes with additional questions on the awareness of BP values and HbA1C measurements. The clinical research section included questions about belief and understanding of clinical research, including enablers and barriers. The survey was piloted at one patient engagement event and finalised after minor revisions. The survey was translated into four main languages (Arabic, Hindi, Somali and Urdu). Translation was carried out collaboratively by the public engagement volunteers comprising lay members, doctors, nurses and pharmacists from the two ethnic minority groups. Feedback on the translated material was obtained from an independent group of volunteers, and their recommendations were considered in the revisions before the translations were finalised for use. The official translation was not used as it was expensive.

### Survey procedures

The survey was carried out at Hindu temples, local SA old age pensioner clubs, Glasgow Central Mosque, African restaurant and Glasgow gurdwaras between November 2019 and December 2019. We organised a total of 11 engagement activities; 9 of them were conducted during normal operations of the mosque, gurdwara and temples for the SA and AFR communities. The other two events are separate events organised one in the gurdwara and one in an African restaurant for SA and AFR populations, respectively. Participants (age > 18 years) of self-identified South Asian or African ancestry who attended these public engagement events were offered to have their blood pressure measured and/or complete the survey. They were offered the option to either complete the survey on-site at the event or take it home to return at a later stage. There were 15–20 volunteers at each event to assist with the completion of the survey—either transcribing responses or reading out items or providing additional translational help. There were no financial incentives associated with the completion of the survey. A total of 500 surveys were distributed.

### Consent and ethical considerations

The survey was opt-in with consent implied through completion of the survey; therefore, no separate consent was obtained. No identifiable information was collected. Permission to distribute the questionnaire was obtained from lay leaders of the community or event organisers.

### Data capture and storage

Data from the paper forms were transcribed by two independent researchers separately (SL, SJ and GD) and then harmonised into an Excel spreadsheet.

### Statistical methods

The sample size was not pre-specified because this was a survey to identify enablers and barriers to participation in clinical research by SA and AFR ethnic minority groups to inform future studies. Continuous variables will be described by mean, standard deviation, range, total number and number of missing data. Categorical variables will be described by frequency counts and percentages in each category. Group comparisons will be made using Student’s *t* test for continuous variables and the chi^2^ test for categorical variables. Statistical analyses were conducted using SPSS (version 27).

### Ethics

Ethics approval was obtained from the MVLS College Ethics Committee, University of Glasgow 200190062.

## Results

### Demographics

Of the 500 surveys distributed, 337 (67.4%) were returned—242 (71.8%) SA and 56 (16.9%) AFR. There were 39 (13%) surveys excluded because they were returned with no questions answered. Majority of respondents from both ethnic groups were not born in the UK (SA = 150 (62.8%), AFR = 51(91.1%)). Twenty-nine (51%) and 113 (47%) AFR and SA participants, respectively, were 35–54 years old, while 4 (7%) and 75 (31%), respectively, were > 55 years. The proportion of participants living in the least deprived (SIMD 1) and most deprived (SIMD 5) areas were 26 (12.4%) and 34 (16.2%), respectively, for SA and 20 (42.6%) and 2(4.3%), respectively, for AFR. A majority of SA and AFR had at least two generations living in the same household. Demographic data are summarised in Table 1.

**Table 1:** Demographics

		South Asian (total ***N*** = 242)		African (total ***N*** = 56)		***P*** values
		***n***	%	***n***	%	
**Gender**	Male	158	65.3%	34	60.7%	0.519
	Female	84	34.7%	22	39.3%	
**Born in the UK**	Yes	89	37.2%	5	8.9%	≤ 0.001
	No	150	62.8%	51	91.1%	
**Age groups**	≤ 18 years old (excluded)	4	1.7%	3	5.3%	0.007
	19–24 years old	15	6.2%	6	10.5%	
	25–34 years old	35	14.5%	15	26.3%	
	35–44 years old	68	28.1%	19	33.3%	
	45–54 years old	45	18.6%	10	17.5%	
	55–64 years old	34	14.0%	4	7.0%	
	65–74 years old	25	10.3%	0	0.0%	
	75 years or older	16	6.6%	0	0.0%	
**SIMD 2020 deciles**	1.00	26	12.4%	20	42.6%	≤ 0.001
	2.00	68	32.4%	12	25.5%	
	3.00	24	11.4%	4	8.5%	
	4.00	58	27.6%	9	19.1%	
	5.00	34	16.2%	2	4.3%	
**Years lived at current address**	< 1 year	26	11.2%	5	9.1%	0.107
	1–3 years	44	19.0%	12	21.8%	
	3–5 years	29	12.5%	14	25.5%	
	> 5 years	129	55.6%	22	40.0%	
	Prefer not to answer	4	1.7%	2	3.6%	
**Number of generations in household**	1	68	28.1%	23	40.4%	0.006
	2	132	54.5%	34	59.6%	
	3	40	16.5%	0	0.0%	
	4	2	0.8%	0	0.0%	
**Employment status**	Employed	110	45.5%	33	57.9%	≤ 0.001
	Carers	5	2.1%	1	1.8%	
	Retired	39	16.1%	0	0.0%	
	Student	12	5.0%	10	17.5%	
	Unemployed/unknown	76	31.4%	13	22.8%	
**Overall happiness with health**	Very happy	183	75.9%	43	78.2%	0.723
	Happy	58	24.1%	12	21.8%	

### Health and well-being

Participants were asked if they had blood pressure or HbA1c measurements conducted in the last year. Blood pressure checks in the previous 12 months were confirmed by 134 (55%) and 25 (44%) of SA and AFR, respectively, and HbA1C checks in 51 (21%) and 10 (18%), respectively. The SA group reported a higher prevalence of heart disease 15 (6%), stroke 5 (2%), hypertension 5 (2%) and diabetes 9 (3.7%) compared to AFR.

### Clinical research

The responses to questions related to clinical research are summarised in Table 2. A majority agreed that different ethnic groups may respond differently to treatments and recognised that treatment needs to be tailored for ethnicity (SA = 107 (48%), AFR = 23 (44.1%)). A majority affirmed that each ethnic group must participate in research studies to find the best treatment for them (SA = 158 (70.9%), AFR = 40 (80%)). Most felt that the current cardiovascular disease treatment guidelines were not tailored for different ethnicities (SA = 84 (38.5%), AFR = 23 (45.1%)).

**Table 2:** Survey responses to clinical research and understanding of health characteristics

		South Asian (total ***N*** = 242)		African (total ***N*** = 56)	
		*n*	%	*n*	%
**Different ethnic groups may respond differently to different treatments, and this means each ethnic group must be part of studies to find the best treatment for them. Do you agree with this statement?**	**Yes**	158	70.90%	40	80.00%
	**No**	30	13.50%	5	10.00%
	**Maybe**	31	13.90%	5	10.00%
	**Prefer not to answer**	4	1.70%	0	0.00%
**Do you think that the current treatment guidelines for cardiovascular diseases are tailored for different ethnicities?**	**Yes**	79	36.20%	10	19.60%
	**No**	84	38.50%	23	45.10%
	**Maybe**	52	23.90%	18	35.30%
	**Prefer not to answer**	3	1.40%	0	0.00%
**Do you believe the treatment of conditions for your ethnic group is likely to be different compared to other ethnic groups?**	**Yes**	107	48.00%	23	45.10%
	**No**	51	22.90%	12	23.50%
	**Maybe**	37	16.60%	16	31.40%
	**Prefer not to answer**	28	12.60%	0	0.00%
**Understanding of waist circumference**	**Yes**	61	25.20%	8	14.00%
	**No**	90	37.20%	27	47.40%
	**Do not know**	91	37.60%	22	38.60%
**Opinion of waist circumference**	**Normal**	43	17.80%	10	17.50%
	**Abnormal**	6	2.50%	1	1.80%
	**Do not know**	193	79.80%	46	80.70%
**Understanding of BMI**	**Yes**	40	16.50%	8	14.00%
	**No**	125	51.70%	29	50.90%
	**Do not know**	77	31.80%	20	35.10%
**Opinion of BMI**	**Normal**	21	8.70%	4	7.00%
	**Abnormal**	10	4.10%	2	3.50%
	**Do not know**	211	87.20%	51	89.50%
**Understanding of blood pressure**	**Yes**	90	37.20%	14	24.60%
	**No**	75	31.00%	20	35.10%
	**Do not know**	77	31.80%	23	40.40%
**Opinion of blood pressure**	**Normal**	57	23.60%	14	24.60%
	**Abnormal**	24	9.90%	1	1.80%
	**Do not know**	161	66.50%	42	73.70%
**Understanding of diabetes**	**Yes**	33	14.50%	3	5.60%
	**No**	195	85.50%	51	94.40%
**Opinion of diabetes**	**Well controlled**	19	7.90%	2	3.50%
	**Poorly controlled**	2	0.80%	0	0.00%
	**Do not know**	221	91.30%	55	96.50%
**Measurement of BP**	**< 1 year**	134	55.40%	25	43.90%
	**1 year**	23	9.50%	4	7.00%
	**> 1 year**	40	16.50%	8	14.00%
	**Missing**	45	18.60%	20	35.10%
**Measurement of HbA1c**	**< 1 year**	51	21.10%	10	17.50%
	**1 year**	16	6.60%	2	3.50%
	**> 1 year**	35	14.50%	5	8.80%
	**Missing**	140	57.90%	40	70.20%

A majority sought information about their health from their doctors (SA = 113 (46.7%), AFR = 26(47.3%)), did not actively search for research studies (SA = 66 (31.3%), AFR = 19 (38%)), wished to know more about clinical trials and were interested in participating (SA = 55 (22.7%), AFR = 16 (28.1%)), happy to participate if it was for a condition they had (SA = 135 (64.9%), AFR = 35 (70.0%)), a condition they do not have but may benefit others (SA = 120 (61.9%), AFR = 33 (64.7%)) or to study new treatment that will benefit future generations (SA = 128 (62.7%), AFR = 35(70.0%)).

A minority of SA participants said they received an invitation to participate in a clinical research study (36, 16%), and the AFR group preferred not to answer the question (47, 94%)

Both ethnic groups indicated that word of mouth from friends/relatives who have participated in clinical studies would be the preferred option for them to consider participating (SA = 112 (46.3%), AFR = 30 (52.6%)), and a greater proportion of AFR than SA would prefer research studies to be conducted in places they frequently visit (SA = 65 (26.9%), AFR = 24(42.1%)).

The enablers and barriers are shown in Table 3. The key enabler that would encourage both groups to participate in research is the provision of information to help them manage their health condition. For the SA group, other enablers include conducting aspects of their clinical research visits/appointments at a location they frequently visited and allowing a family member to attend the clinical research visit to help with the decision-making process. Barriers that were similar in both groups included concerns about how personal information will be used, who will have access to personal data (SA = 54 (22.4%), AFR = 18(31.6%)) and whether side effects of the new treatment may be worse than current treatment (SA = 53 (22.0%), AFR = 16 (21.8%)). After completion of the questionnaire, a majority said that they would consider participating in clinical research in the near future (SA = 110 (45.5%), AFR = 39 (64.4%)).

**Table 3:** Enablers and barriers of participation of ethnic minorities in clinical research

	South Asian (total ***N*** = 242)		African (total ***N*** = 56)	
	***n***	%	***n***	%
**Enablers**				
Being provided with supporting information on managing my health condition in general.	81	33.6%	25	43.9%
Conducting certain aspects of your clinical research visits/appointments at a more convenient location (i.e. places you frequently visit—local religious places of worship).	64	26.6%	20	35.1%
My family member can come along with me to the visit to help me in the decision-making process.	47	19.5%	10	17.5%
Monetary incentive.	44	18.3%	10	17.5%
I will be able to help save or improve the lives of patients with a similar condition.	40	16.6%	21	36.8%
Other reasons.	35	14.5%	8	14.0%
If I would have access to the study drug after my participation ended.	32	13.3%	10	17.5%
Clinical trial mobile application (to remind me of appointments, patient information sheets, my current progress in the clinical trial, etc.).	45	18.7%	7	12.3%
I will be seeing a specialist of my medical condition as part of my clinical trial visits.	37	15.4%	6	10.5%
Reduce the need to see my GP.	36	14.9%	15	26.3%
It will help advance the science and the treatment of my disease or condition.	30	12.5%	6	10.5%
Prefer not to answer.	16	6.7%	0	0.0%
**Barriers**				
I am concerned about how my personal information will be used and who will have access.	54	22.4%	18	31.6%
I am worried that I am being used for research without any benefit for me.	19	7.9%	6	10.5%
I do not trust people who carry out research.	8	3.3%	1	1.8%
I find it difficult to speak to doctors and nurses.	16	6.6%	4	7.0%
I have not heard anyone in my community taking part in the trial, so it must not be relevant to me.	35	14.5%	9	15.8%
The side effects may be worse than my current treatment.	53	22.0%	16	28.1%
If I take part in a trial, my disease may get worse.	29	12.1%	6	10.5%
I will be exposed to a lot of unnecessary investigations that may have no benefit.	26	10.8%	8	14.0%
Religious views.	8	3.3%	1	1.8%
Other reasons.	21	9.1%	0	0.0%
Prefer not to answer.	55	24.3%	6	10.5%

## Discussion

In our hypertension-focused survey of South Asian and African adults in Glasgow, we find that a majority had had their blood pressure checked in the previous 12 months despite reporting a lack of understanding of their blood pressure or hypertension status. This view was similar for diabetes as well. We find high levels of reported interest in understanding personal health and recognition of the importance of research and participation in research studies. We confirm previous findings about enablers of trial participation including referrals from friends or relatives who had previously participated in the trials, conducting trial visits to places they frequently visit, and research studies on a condition they suffer from among both AFR and SA survey participants [7, 13]. The gender distribution of both the SA and AFR was skewed towards majority male participation (65% males among SA and 61% among AFR). Most of the events that we organised were in religious venues where there were no restrictions on male and female attendances. We had a separate female-only volunteer group to screen female mosque attendees. However, it is clear that this is not adequate to ensure greater female engagement and more studies on this are warranted. The greater proportion of SA compared to AFR participants in our study population reflects the ethnic minority demographics of the Greater Glasgow area. The 2011 UK census found that 15% of the population of Glasgow belonged to an ethnic minority group, and of these, 8.1% and 2.4% were of South Asian and African ancestries, respectively. For the AFR community, only one event was organised in a restaurant for AFR populations attended by < 100 individuals which explains the differences in numbers between the two groups.

The difficulties in recruiting research participants in general are well recognised. The key enablers [14, 15] that motivate participants are altruism, an expected personal gain in terms of specialist review/investigations or payments and perceived additional health benefits while the barriers [14–17] include a lack of trust in the health profession/research staff, concerns about data confidentiality, inconvenience or discomfort, lack of access to information about the study, lack of access to clear information, stigma of inclusion in research and a reluctance to take part in higher risk studies. The disproportionate underrepresentation of ethnic minorities in research studies is likely due to all these factors, plus additional barriers related to trial burden, language support, perceived discriminatory practices, mistrust of health care personnel and ‘legacy of exploitation’ [14–16, 18]. The lack of ethnic minority representation in research limits the generalisability of results that affects the allocation of resources for services and research and deprives people of ethnic minority groups to benefit from the best treatments [19].

Our survey does not identify any novel barriers that deter ethnic minority participation in research. However, our survey has a lot of positive and encouraging signals that indicate a route to enhancing ethnic minority engagement with research. Our response rate of 67% is very high and reflects the value of this personalised approach with engagement activities carried out in an environment that are convenient and acceptable to each community. We included volunteers from the ethnic minority groups to help overcome the language barriers. We had the support of community leaders who promoted the event and encouraged participation. For the research questionnaire, we had research nurses and doctors who were able to explain and demystify the research process. Although 337 surveys were completed, the number of people who had their BP measured was considerably higher especially in males, and this may reflect the higher prevalence of hypertension among males compared to females. The survey team did not record BP that was in the normal range for those who did not complete the survey. However, we had all abnormal BP measurements recorded irrespective of survey completion and every initial high BP reading required an additional 2 measurements for confirmation, and this resulted in a note to the GP. There were 262 individuals (SA/AFR) with undiagnosed high blood pressure across all the events, and we observed a number of women attending mosques who had never seen a doctor before had high BP readings. When some of the participants declined to have their BP assessed by their GP or even consider treatment, we obtained support from the mosque leaders who were very proactive in advising the persons to seek help and disabuse their belief that religion will protect them from hypertension outcomes. The unrestricted support we obtained from the community and religious leaders reflects a good grasp of the value of research and especially the health and social benefits to the community and highlights the evolution of beliefs and fears among thought leaders within the community. The responses to the questionnaire indicate that minimising trial burden on the participants by moving the trial to the participant rather than bringing the participant to the trial is essential. The high response rate offers high confidence in the representativeness of our findings.

This was a very resource-intensive exercise involving a large team of volunteers, logistics organisation, transport of equipment and informational material and multiple meetings with community leaders and representatives. The opportunistic sampling of participants who attended the public engagement events may limit generalisation to the wider respective populations who do not ordinarily attend such activities or places or limit the application of our findings to other ethnic groups and to other cities.

Future research should focus on understanding enablers and barriers in a range of communities such as those living in severe and enduring poverty, those from minority ethnic groups, those with experience of seeking asylum and of homelessness and those engaging participants across the life course. Research into methods to promote public dialogue among marginalised and underserved groups will develop our knowledge of how to bring patients, the public and health care professionals together to address representation in clinical research studies. Research in methods of public dialogue that allows people to connect to each other (digitally or face-to-face, synchronously or asynchronously) to exchange ideas and perspectives and how community leaders and other representatives can enhance and improve the effectiveness of public dialogue. Other areas of research include investigating the acceptability of virtual trials, remote monitoring, digital trials and newer adaptive trial designs including platform trials that have recently demonstrated their strengths and value during the COVID pandemic.

In conclusion, our hypertension-focused survey provided some insights into our poor recruitment of ethnic minorities despite our prior one-size-fits-all patient and public engagement activities. It informs local researchers about possible key enablers which will encourage the recruitment of ethnic minorities local into clinical trials. Our study highlights the importance of community education, specifically understanding the health characteristics and secondly the crucial impact of the community leaders (such as mosque leaders) in such education and promotion of the research trials. We have identified that adaptations to public engagement activities are required to ensure it reaches all individuals especially in a diverse locality in Glasgow. Our survey reinforces the need for continual review of researchers’ beliefs and attitudes, as patient and community leaders’ awareness and beliefs are rapidly evolving for the better, and this requires adaptations of engagement and recruitment activities to keep up with these changing perceptions.

## Supplementary Information

**Additional file 1.** Survey of awareness and attitudes to clinical research and cardiovascular health among ethnic minorities in Glasgow.

## Data Availability

Not applicable

## Abbreviations

AFR: African
BP: Blood Pressure
GBPC: Glasgow Blood Pressure Clinic
HbA1c: Glycated hemoglobin test
SA: South Asian
UK: United Kingdom
