# Supplementary material for: Underrepresentation of ethnic minorities in hypertension research—a survey of enablers and barriers among South Asian and African communities in Glasgow
Source: Trials. 2022 Jul 29;23:609. doi: 10.1186/s13063-022-06542-z (PMC9335986; doi:10.1186/s13063-022-06542-z)
Supplement: Supplementary file 1 — Additional file 1. Survey of awareness and attitudes to clinical research and cardiovascular health among ethnic minorities in Glasgow. [file 13063_2022_6542_MOESM1_ESM.pdf]

University of Glasgow | College of Medical, Veterinary & Life Sciences

Survey of awareness and attitudes to clinical research and cardiovascular health among ethnic minorities in Glasgow

Dr Stefanie Lip, Dr Linsay McCallum, Dr Katriona Brooksbank, Miss Aishah Mahmud, Dr Nazim Ghouri, Professor Sandosh Padmanabhan.

Please read the participation information sheet before filling out the survey

Ethics approved, application number: 200190062((date:251119)

Survey of awareness and attitudes to clinical research and cardiovascular health among ethnic minorities in Glasgow

Participant Information Sheet

This participation information sheet is a guide to the questions in the survey. There will be no participant identifiable information collected. Please take your time to read the questions and information sheet.

Section 1 Demographics

This section is to find out which ethnic group you belong to, when you arrived in the United Kingdom, your age, your gender, employment status and how many live in the same household.
We ask for your postcode which will allow us to look at the Scottish Index of Multiple Deprivation. This will provide us with data across seven domains: current income, employment, health, education, skills and training, housing, geographic access and crime. Please bear in mind that this will not provide us with any identifiable information on where you live, employment or income status.

Section 2 Health and Wellbeing

This section is to find out if you have been diagnosed with any medical conditions, please feel free to tick more than one answer and also rate how happy you are with your health

Section 3: Clinical Research

We would like to know your thoughts and understanding about clinical research. We are not trying to recruit you into a research study. We are also interested in your thoughts and experiences about previous/current participation in medical research studies. We would like to hear your thoughts, questions, concerns, beliefs and experiences.

Thank you for participating in this survey

Section 1: Demographics

Which of the following ethnic groups do you belong to?

☐ White Scottish<br>☐ White Irish<br>☐ Other White British<br>☐ Any other White background	☐ Indian<br>☐ Pakistani<br>☐ Bangladeshi<br>☐ Other South Asians	☐ African-Caribbean<br>☐ African<br>☐ Black Scottish or any other Black background	☐ Chinese<br>☐ Any Mixed Background<br>☐ Any Other Background	☐ Prefer not to answer

Were you born in the United Kingdom?

☐ Yes	☐ No	☐ Prefer not to answer

What year did you first come to live in the United Kingdom (if applicable)?

Year _________	☐ Do not know	☐ Prefer not to answer

Which of the following age groups do you belong to?

☐ <18 years old<br>☐ 18 - 24 years old	☐ 25 - 34 years old<br>☐ 35 - 44 years old	☐ 45 - 54 years old<br>☐ 55 - 64 years old	☐ 65 - 74 years old<br>☐ 75 years or older
☐ Do not know		☐ Prefer not to answer	

I identify myself as

☐ Male	☐ Female	☐ Other___________	☐ Prefer not to answer

First three characters of postcode: ______________ ☐ Prefer not to answer

How long have you stayed at this current address?

☐ < 1 year	☐ 1 – 3 years	☐ 3 – 5 years	☐ > 5 years
☐ Do not know		☐ Prefer not to answer	

Including yourself, how many people are living together in your household? *(Include those who usually live in the house such as students living away from home during term, partners in the armed forces or professions such as pilots)*

☐ Enter number ______________	☐ Do not know	☐ Prefer not to answer

How are the other people who live with you related to you? *(You can select more than one answer)*

☐ Husband, wife or partner<br>☐ Son and/or daughter (include step-children)	☐ Brother and/or sister<br>☐ Mother and/or father	☐ Grandparent<br>☐ Grandchild	☐ Other related<br>☐ Other unrelated
☐ Do not know		☐ Prefer not to answer	

Which of the following describes your current situation? (*You can select more than one answer)*

☐ In paid employment - shift work including night shifts<br>☐ In paid employment - shift work with no night shifts<br>☐ In paid employment - no shift work	☐ Self-employed<br>☐ Unable to work because of sickness or disability<br>☐ Unemployed	☐ Retired<br>☐ Looking after home and/or family<br>☐ Doing unpaid or voluntary work
☐ Full or part-time student	☐ Carer<br>☐ On maternity leave	☐ None of the above
☐ Do not know	☐ Prefer not to answer	

Section 2: Health and Wellbeing

In general, how happy are you with your overall health? *(Tick one)*

☐ 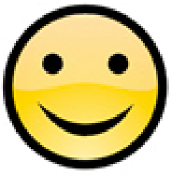	☐ 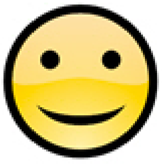	☐ 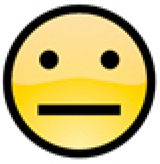	☐ 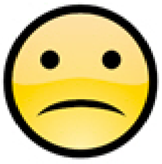	☐ 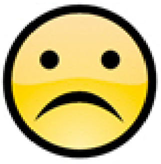

Do you know what your waist circumference is and what it means?

☐ Yes<br>It is ____________ cm / inches<br>☐ Normal ☐ Abnormal ☐ Don't Know	☐ No	☐ Prefer not to answer

Do you know what your BMI is and what it means?

☐ Yes<br>It is ____________ cm/m^2<br>☐ Normal ☐ Abnormal ☐ Don't Know	☐ No	☐ Prefer not to answer

Has your doctor ever told you that you have had any of the following medical conditions? (You can select more than one answer)

Select from

☐ Heart disease<br>☐ Stroke<br>☐ Severe depression	☐ Chronic bronchitis / emphysema<br>☐ Alzheimer's disease / dementia<br>☐ Parkinson's disease	☐ Lung cancer<br>☐ Bowel cancer<br>☐ Prostate cancer	☐ Other<br>____________________<br>☐ None of the above<br>☐ Do not know<br>☐ Prefer not to answer

Do you know your Blood Pressure is and what it means?

☐ Yes<br>It is _____/______ mmHg<br>☐ Normal ☐ Abnormal ☐ Don't Know	☐ No	☐ Prefer not to answer
**My blood pressure was last measured**		
☐ < 1 month ago<br>☐ 1 – 3 months ago<br>☐ 4 – 6 months ago	☐ 6 – 12 months ago<br>☐ 1 year ago<br>☐ > 1 year	☐ Never ever been measured<br>☐ Prefer not to answer

Do you have Diabetes?

☐ Yes<br>It is<br>☐ Well controlled ☐ Poorly controlled ☐ Don't know	☐ No	☐ Prefer not to answer
**When was your blood glucose or HbA1c checked?**		
☐ < 1 month ago<br>☐ 1 – 3 months ago<br>☐ 4 – 6 months ago	☐ 6 – 12 months ago<br>☐ 1 year ago<br>☐ > 1 year	☐ Never been measured<br>☐ Prefer not to answer

Section 3: Clinical Research

Clinical research plays an important role in developing treatment guidelines for medical conditions.

Different ethnic groups may respond differently to different treatments and this means each ethnic group must be part of studies to find the best treatment for them.
Do you agree with this statement?

☐ Yes	☐ No	☐ Maybe	☐ Prefer not to answer

Do you think that the current treatment guidelines for cardiovascular diseases are tailored for different ethnicities?

☐ Yes	☐ No	☐ Maybe	☐ Prefer not to answer

Do you believe treatment of conditions for your ethnic group is likely to be different compared to other ethnic groups?

☐ Yes	☐ No	☐ Maybe	☐ Prefer not to answer

Where do you search for your information about health for yourself or for somebody you care about?

☐ Internet<br>☐ NHS patient information sites eg. NHS 24	☐ Speaking to your GP<br>☐ Speaking to your specialist doctor	☐ Friends / Family<br>☐ Other_________________<br>☐ Prefer not to answer

And if so, would you search for research studies that are being conducted locally?

☐ Yes	☐ No	☐ Prefer not to answer

Have you ever been invited to be in a clinical research study?

☐ Yes	☐ No	☐ Prefer not to answer

Which of the following describes your understanding of clinical trials or research? *(Tick all that apply)*

☐ I do not know anything about clinical trials – I am not interested in knowing more	☐ I do not know anything about clinical trials – I am interested in knowing more	☐ I believe trials are experiments that do not benefit me
☐ I know what clinical trials are and I am keen to participate	☐ I don’t know much about clinical trials, but I am keen to volunteer to help with anything that will benefit others	☐ Prefer not to answer

If you were offered to take part in a trial, would you take part if it is for

A condition that you have?

☐ Yes	☐ No	☐ Prefer not to answer

A condition that you do not have but may benefit others?

☐ Yes	☐ No	☐ Prefer not to answer

A trial to study new treatment which you do not have but may benefit future generations?

☐ Yes	☐ No	☐ Prefer not to answer

Which of the following methods will encourage you to participate in research into medical conditions? *(Tick all that apply)*

☐ Word of mouth from friends / relatives who have taken part	☐ Newspaper advert / radio advert	☐ The research study happening in a place I frequently visit	☐ Other _______________
☐ Leaflets/flyers at your local grocery store / take away / restaurant?	☐ Social media platforms (Facebook, Twitter, Instagram)	☐ Personally approached at public engagement /health awareness events	☐ Prefer not to answer

Usually research participants have their travel costs covered and get a cup of tea and biscuit(s) during their visit. Is there anything else mentioned below that may encourage you to take part? *(Tick all that apply)*

☐ Being provided with supporting information on managing my health condition in general	☐ If I would have access to the study drug after my participation ended
☐ Conducting certain aspects of your clinical research visits/appointments at a more convenient location (ie places you frequently visit – local religious places of worship)	☐ Clinical trial mobile application (to remind me of appointments, patient information sheets, my current progress in the clinical trial etc)
☐ My family member can come along with me to the visit to help me in the decision-making process	☐ I will be seeing a specialist of my medical condition as part of my clinical trial visits
☐ Monetary incentive	☐ Reduce the need to see my GP
☐ I will be able to help save or improve the lives of patients with the similar condition	☐ It will help advance the science and the treatment of my disease or condition
☐ Other reasons: ________________________	☐ Prefer not to answer

Which of the following will hinder my participation in clinical research/trials?
(Tick all that apply)

☐ I am concerned about how my personal information will be used and who will have access.	☐ The side effects may be worse than my current treatment
☐ I am worried that I am being used for research without any benefit for me.	☐ If I take part in a trial my disease may get worse
☐ I do not trust people who carry out research	☐ I will be exposed to a lot of unnecessary investigations that may have no benefit
☐ I find it difficult to speak to doctors and nurses	☐ Religious views
☐ I have not heard anyone in my community taking part in the trial so it must not be relevant to me.	☐ Other reasons:______________________
	☐ Prefer not to answer

Would you consider taking part in a clinical research study in the near future?

☐ Yes	☐ No	☐ Prefer not to answer

Are there any groups or individuals who you think will benefit from this survey?

____

Thank you for your participation
